# On the utility of near-infrared spectroscopy-derived measures for assessing cerebrovascular autoregulation: results from an observational cohort study

**DOI:** 10.1007/s10877-025-01399-4

**Published:** 2025-12-15

**Authors:** Stefan Y. Bögli, Cameron Smith, Ihsane Olakorede, Michal M. Placek, Gemma Bale, Peter Smielewski

**Affiliations:** 1 Brain Physics Laboratory, Division of Neurosurgery, Department of Clinical Neurosciences, University of Cambridge, Cambridge, UK; 2 Neuro Optics Lab, Division of Electrical Engineering, Department of Engineering, University of Cambridge, Cambridge, UK; 3 Department of Neurology and Neurocritical Care Unit, Clinical Neuroscience Center, University Hospital Zurich, University of Zurich, Zurich, Switzerland; 4 Department of Physics, University of Cambridge, Cambridge, UK; 5 Department of Engineering, University of Cambridge, Addenbrooke’s Hospital, Trumpington St, Box 167, Cambridge, Cambridge, UK

**Keywords:** Cerebrovascular autoregulation, Cerebral blood flow, Multimodality neuromonitoring, Near infrared spectroscopy, Traumatic brain injury

## Abstract

Cerebrovascular autoregulation maintains stable cerebral blood flow by counteracting slow changes in cerebral perfusion pressure (termed “slow waves”). Conventional assessment involves invasive techniques using intracranial pressure (ICP) or technically challenging cerebral blood flow velocity (FV) measurements. Near-infrared spectroscopy (NIRS) has emerged as a non-invasive alternative; however, its ability to accurately capture the slow-wave oscillations fundamental to cerebrovascular autoregulation remains uncertain. 412 h of simultaneous ICP, FV, NIRS, and arterial blood pressure (ABP) monitoring from 35 traumatic brain injury patients were explored. Coherence, gain, and Granger causality analyses were employed to assess whether NIRS adequately reflects slow waves in ABP, FV, or ICP to investigate whether NIRS is a suitable alternative for assessing the state of cerebrovascular autoregulation In this single-centre observational cohort study, 89 recordings from 35 moderate to severe traumatic brain injury (TBI) patients (totalling 412 h of artefact-free data) were analysed. Simultaneous high-resolution recordings of NIRS, ICP, FV, and arterial blood pressure (ABP) were acquired. Coherence and gain were computed across defined frequency bands (0.001–0.5 Hz), with a focus on the range most relevant to cerebrovascular autoregulation (0.005–0.05 Hz). Granger causality was used to explore directional relationships between physiological inputs (ABP, FV, ICP) and NIRS outputs (rSO2 and haemoglobin metrics). Haemoglobin-based NIRS metrics (total, oxy-, deoxy-, and delta haemoglobin) demonstrated significantly higher coherence and Granger causality with FV and ICP compared to rSO2 (*p* < 0.001, large effect sizes) capturing the slow-wave oscillations central to cerebrovascular autoregulation. In contrast, rSO₂ exhibited poor coherence and low causality, especially with ABP, likely due to device-specific post-processing and resolution limitations. NIRS derived haemoglobin metrics reliably capture slow-wave dynamics reflective of cerebrovascular autoregulation and reactivity, offering a non-invasive alternative to traditional methods. Conversely, rSO2 lacks sufficient temporal fidelity to detect these fluctuations under routine clinical conditions, limiting its utility for cerebrovascular autoregulation assessment.

**Supplementary Information:**

The online version contains supplementary material available at 10.1007/s10877-025-01399-4.

## Introduction

Cerebrovascular autoregulation is a physiological mechanism of the brain that attempts to maintain stable cerebral blood flow by adjusting the arteriolar diameter to account for slow changes (termed “slow waves”) in cerebral perfusion pressure (CPP) [1, 2]. If this mechanism fails, passive variations in cerebral blood flow associated with hyper- or hypoperfusion occur [3]. At the bedside, cerebrovascular autoregulation may be assessed by measuring intracranial pressure (ICP) – to estimate the pressure reactivity index [4] – or cerebral blood flow velocity (FV) – to estimate the mean flow index [5]. These indices quantify two distinct measures, each influenced by changes in arteriolar diameter, but exhibiting different responses to alterations in cerebral physiology [6–8]. The mean flow index quantifies cerebrovascular autoregulation since it directly quantifies the change in flow velocity, while the pressure reactivity index quantifies cerebrovascular reactivity, which quantifies to what extent the arterioles constrict or dilate in response to the cerebrovascular autoregulation mechanism [7, 9]. Since both methods require the placement of an invasive pressure transducer and the mean flow index additionally necessitates transcranial Doppler monitoring, near infrared spectroscopy (NIRS) has been explored as a potential alternative [10–15]. NIRS (e.g. Masimo Root [16, 17], Medtronic INVOS [17, 18], Hamamatsu NIRO [17, 19, 20]) is a widely used, non-invasive long-term monitoring method. NIRS quantifies relative changes in oxy-, and deoxyhaemoglobin, thereby allowing for the estimation of regional oxygen saturation (rSO2), and total (oxyhaemoglobin + deoxyhaemoglobin) and delta haemoglobin (oxyhaemoglobin - deoxyhaemoglobin). Currently, the rSO2 derived cerebral oxygenation index is assumed to reflect blood flow autoregulation (akin to mean flow index) due the association between rSO2 and cerebral blood flow, while the total haemoglobin index (derived from total haemoglobin) is assumed to represent vascular reactivity (equivalent to the pressure reactivity index) due to its association to blood volume [15, 21, 22]. 

When quantifying cerebrovascular autoregulation using correlation coefficients such as the cerebral oxygenation index, it is essential to recognise that correlation coefficients can be derived from any set of signals that exhibit some degree of correlation. While earlier research largely calculated NIRS-derived autoregulation indices without questioning the underlying assumptions, more recent studies have raised concerns about whether the slow waves, which are fundamental to cerebrovascular autoregulation quantification, are adequately captured by NIRS [13, 16, 23, 24]. Therefore, our objective was to evaluate this key prerequisite – specifically, whether NIRS captures the slow waves relevant to cerebrovascular autoregulation, which metric best reflects these dynamics, and to what extent they are reflective of cerebrovascular autoregulation or reactivity.

## Materials and methods

This monocentre cohort study was conducted in the Neurocritical Care Unit, Addenbrooke’s Hospital, Cambridge University Hospital NHS Foundation Trust, University of Cambridge. Consecutive patients admitted between March 2022 and 2024 due to TBI were evaluated. The inclusion criteria were: (1) Age ≥ 18; (2) Acute TBI; (3) Invasive ICP monitoring. The exclusion criteria were: (1) Patient incompatible with transcranial Doppler probe holder placement (e.g. penetrating TBI, severe skull fractures or wounds) or with insufficient bone window for extended monitoring; (2) Patient in preparation for extubation or undergoing withdrawal of life-sustaining measures. The decision for ICP monitoring follows a local adaptation [25] of the Brain Trauma Foundation guidelines [26]. All patients were sedated and ventilated during the recordings. The sample size was determined based on the patients’ availability and the practical constraints, including the necessary resources for transcranial Doppler monitoring. The study followed the principles outlined in the Declaration of Helsinki. The data was acquired as part of the Brain Physics database, which was approved by the local ethics committee (REC 23/YH/0085). Transcranial Doppler was performed as part of a clinical audit (Clinical Project ID4201).

### Data acquisition

A comprehensive overview of the monitoring, including details of the dataset, can be found here [27]. The following monitors were used: (1) The Masimo Root device with NIRS O3 regional oximetry (Masimo Corporation, USA) – attached to both sides of the patients’ forehead, allowing for the acquisition of rSO2 and the haemoglobin metrics (total, oxy, deoxy, and delta haemoglobin concentrations); (2) The DWL (Compumedics, Germany) and Delica (Shenzhen Delica Medical Equipment Ltd, China) transcranial Doppler devices, used for bilateral monitoring of middle cerebral artery FV; (3) The ICP monitor (CereLink) using intraparenchymal sensors (Codman & Shurtleff, USA). In addition, ABP was measured using pressure sensors (Transpac IV Pressure Transducer, ICU Medical, USA) attached to arterial lines (Baxter Healthcare, Illinois, USA)) inserted into the radial or femoral artery, zeroed at the level of the third ventricle, and streamed through bedside monitors (CARESCAPE B650 Monitor, GE Healthcare, USA). Full waveform resolution data was streamed from the separate devices to a laptop and recorded using the ICM + software (Cambridge Enterprise Ltd, UK) [28]. The sampling rate of the NIRS device was 1 Hz and 100 Hz (or higher) for all the other devices. To minimise information bias, data collection was standardised using the same protocols across all patients and devices. The NIRS parameters were specifically kept the same for all recordings. ICM + was responsible for device synchronization between devices.

### Data preprocessing and statistical analysis

Data was pre-processed (as previously described to remove sections of artefactual origin before further processing [27]) and processed within the ICM + software to perform the subsequent signal processing. Statistical analysis and figure preparation were performed using R Studio (R version 4.4.1 - https://www.r-project.org/ - packages used: *tidyverse*,* gtsummary*,* rstatix*,* ggplot2*). For the NIRS metrics and FV, all the described calculations were first performed for each side separately and then averaged point-by-point. For statistical analysis, one summary value per recording was considered. To address researcher bias, we performed various analyses, all aiming at addressing the same question using different methods. Additionally, standardised frequency ranges were used for exploration. Wilcoxon rank sum tests were used for comparison. The resulting p-values were adjusted using the Bonferroni correction to account for multiple comparisons. Additionally, the effect size is reported for significant differences to quantify the magnitude of the differences identified. These are interpreted according to Cohen’s conventional thresholds (small ≥ 0.1, moderate ≥ 0.3, large ≥ 0.5) [29].

### Frequency bands explored

All frequency bands explored are described in Table 1. The initial analysis was performed considering eight frequency bands spanning from 0.001 to 0.5 Hz, with band boundaries defined on a logarithmic or linear scale, allowing to focus on lower (logarithmic scale) or higher (linear scale) frequencies. We performed additional explorations considering either the frequency band that corresponds to the range represented by the pressure reactivity and mean flow index calculations (Brain Physics Lab – BPL, 0.005 to 0.05 Hz) [30] or the frequency bands proposed by the CARNet (Cerebral Autoregulation Research Network) White Paper [31, 32] (frequency bands in Hz: very low 0.02 to 0.07; low 0.07 to 0.2; high 0.2 to 0.5). Importantly, cerebrovascular autoregulation can only counteract slow changes in blood pressure, and is most effective at frequencies below 0.05 Hz [31, 33]. This limitation arises from the time required for arterioles to dilate or constrict in response to pressure fluctuations. Frequencies above 0.07 Hz likely do not provide meaningful information about the state of cerebrovascular autoregulation [34–38]. In line with the stated aims, the results and discussion will largely focus on the slower frequencies represented by the BPL range and the very low frequency range. The other ranges have been explored for completeness and future reference.

**Table 1:** Frequency bands

Logarithmic Scale		Linear Scale			Autoregulation		
Label	Frequency Band (Hz)	Label	Frequency Band (Hz)		Label	Frequency Band (Hz)	
Lg 1	0.001 to 0.002	Ln 1	0.001 to 0.063		BPL	0.005 to 0.05	
Lg 2	0.002 to 0.004	Ln 2	0.063 to 0.126		ULF	0.001 to 0.02	
Lg 3	0.004 to 0.01	Ln 3	0.126 to 0.188		VLF	0.02 to 0.7	
Lg 4	0.01 to 0.02	Ln 4	0.188 to 0.251		LF	0.07 to 0.2	
Lg 5	0.02 to 0.04	Ln 5	0.251 to 0.313		HF	0.2 to 0.5	
Lg 6	0.04 to 0.1	Ln 6	0.313 to 0.376				
Lg 7	0.1 to 0.2	Ln 7	0.376 to 0.438				
Lg 8	0.2 to 0.5	Ln 8	0.438 to 0.5				

* Different frequency bands were explored between 0.001 and 0.5 Hz. Specifically, these included in a first step a logarithmic and linear scale, allowing to focus on lower frequencies and higher frequencies, respectively. Additionally, we explored the Brain Physics Lab (BPL) range (0.005 to 0.05 Hz) that approximately corresponds to the range assessed by the pressure reactivity and mean flow indices and the frequency bands proposed by the CARNet (Cerebral Autoregulation Research Network) White Paper (including the very low, low, and high frequency bands corresponding to 0.02 to 0.07 Hz, 0.07 to 0.2 Hz, and 0.2 to 0.5 respectively). Importantly, cerebrovascular autoregulation has its greatest effectiveness when incoming slow waves have a frequency around 0.05 Hz. The faster frequencies beyond 0.07 reflect other physiological mechanisms and provide little meaningful information about the state of cerebrovascular autoregulation

*Abbreviations: CARNet - Cerebral Autoregulation Research Network; Lg – logarithmic scale; Ln – linear scale; BPL – Brain Physics Lab; HF – high frequency; LF – low frequency; VLF – very low frequency; ULF – ultra low frequency

### Coherence and gain

Coherence and gain were calculated using moving windows of 1200 s with 95% overlap. Any 1200-second data window with missing data was rejected from further analysis. Power spectral estimation was performed using the Welch method (i.e. five windows with 50% overlap). These metrics were derived using standardised methodology [32]:

1. Coherence [39, 40] – Coherence was calculated as the magnitude of the cross-spectrum divided by the product of the power spectra for ABP, ICP, or FV and the NIRS metrics. The maximum coherence within the relevant frequency band was extracted as the summary measure. From a practical standpoint, coherence assesses the relationship between two signals in the frequency domain (after Fourier decomposition into independent sinusoidal components). When coherence is close to 1 for a specific frequency, it means the two signals follow each other in a consistent, linear way, at that frequency. On the contrary, if coherence is close to 0, it suggests there’s little to no relationship between them at that frequency. Very low coherence can also result from noise, significant non-linear effects, or other confounding variables which affect the NIRS metric [39, 40]. Sufficient coherence was defined based on the CARNet White paper as 0.34. [31]

2. Gain [39, 40] – Gain was estimated using the transfer function for the same time windows and signals as described above. Gain quantifies the extent to which changes in one signal are reflected in another – i.e. it measures the strength of the response, and it is also a function of frequency. High gain within a specific frequency range suggests that fluctuations in ABP (or another input) are strongly transmitted to the NIRS metric. Gain itself does not have a cutoff since it depends on the absolute values of signals analysed as well as the state of autoregulation.

These metrics were first explored within the logarithmic and linear ranges to see frequency-specific dynamics. In a second step, further analyses were performed considering only the BPL range. First, dot plots were explored to assess whether relevant outliers (specifically clusters with exceptionally high or low coherence or gain of any of the NIRS metrics to either ABP, ICP or FV) exist. Second, we explored whether high vs. low (upper vs. lower third) ABP slow wave power (i.e. the ‘strength’ of the challenge to the cerebrovascular autoregulation mechanism) would affect the coherence between the different metrics using Wilcoxon rank sum tests. Third, to assess the strength of association, we conducted Wilcoxon rank-sum tests to identify which NIRS-derived signals showed the highest coherence with each of the ABP, FV, or ICP signals. Specifically, we performed two comparisons: (1) comparing different NIRS metrics to a single input signal to determine which NIRS metric displayed the highest coherence with that input, and (2) comparing a single NIRS metric to the different input signals to identify which input exhibited the highest coherence with that specific NIRS metric. Part of these analyses were repeated for the CARNet ranges to allow for a fuller picture of the influence of the different frequency ranges.

### Granger causality

Lastly, we applied Granger causality, [41] which captures whether one time series (in our case ABP, FV, or ICP) can help predict future values of another time series (in our case the NIRS metrics), beyond what the latter’s past values alone can predict. We compared their respective causal strengths as indicated by F statistics. Given our hypothesis that changes in NIRS metrics represent downstream effects of FV or ICP alterations, we also examined the Granger causality between ABP and FV or ICP. For all Granger causality analyses, differencing was applied to enforce stationarity, and the influence of lag in information transfer was determined based on the Bayesian Information Criterion with a delay limit of 10. From a practical perspective, a significant Granger causal relationship suggests that changes in ABP (or another input) precede and contribute to subsequent changes in the NIRS metrics. The presence of a temporal delay is essential for valid assessment of autoregulation, since the change in arteriolar adjustments occurs over time with physiologically plausible response times ranging from 1 to 10 s [35, 42]. Of note, Granger causality – even when statistically significant – does not imply true physiological causation (i.e., that changes in the output variable are directly caused by changes in the input variable). Rather, it reflects a directional predictive relationship, suggesting a potential causal link. In this analysis, we employed Granger causality to assess the relative strength of associations between various input and output metrics, thereby identifying which input best predicts its corresponding output without relying on predefined thresholds.

## Results

**Fig. 1:**
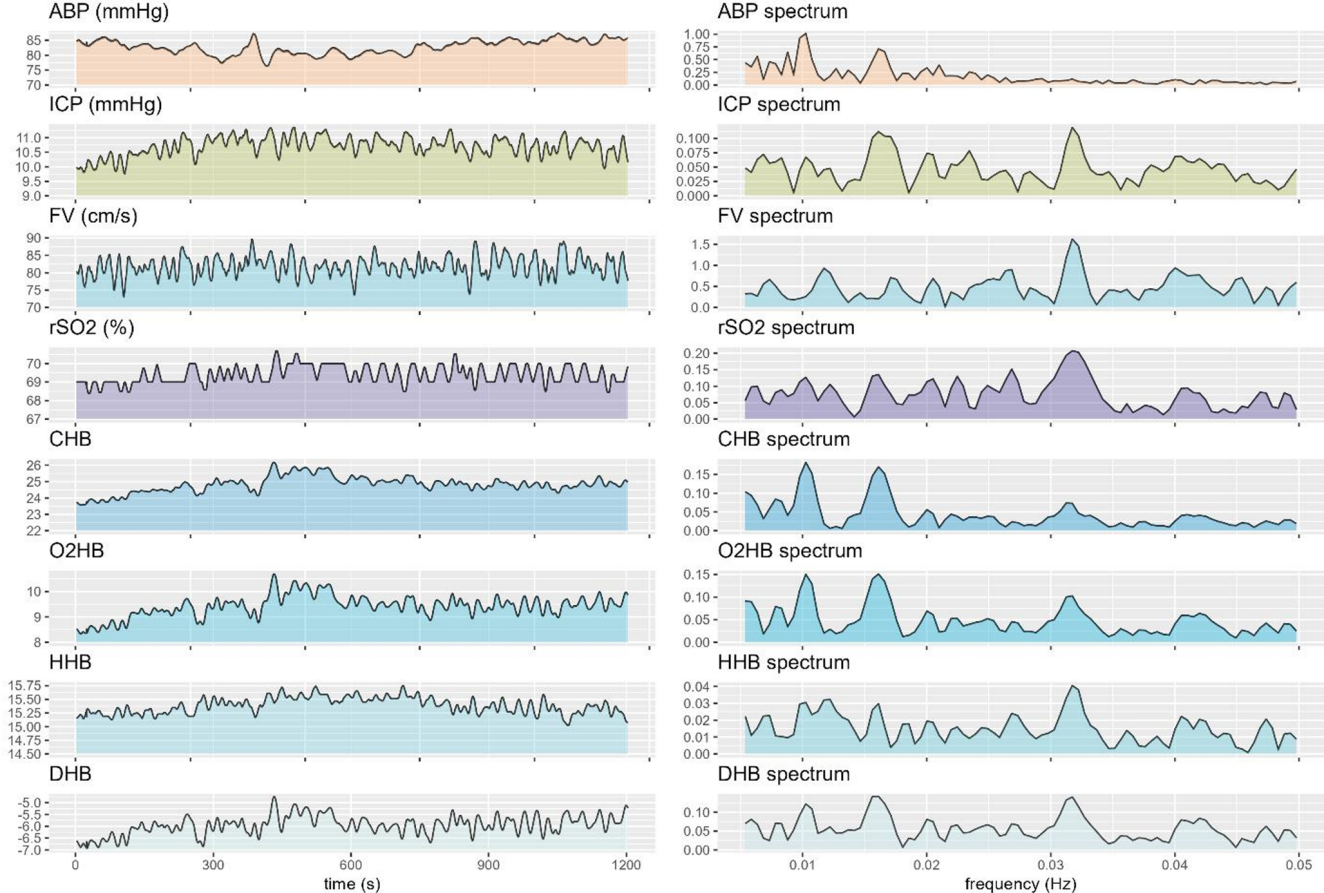
Raw metrics and corresponding frequency spectrum charts.* Example time trends of the raw metrics (left charts) are displayed in comparison with the derived spectrum (0.005 to 0.05 Hz) 30 exemplifying the distinct differences between the raw metrics as well as the derived spectrum charts. Due to the post-processing applied by the NIRS device for the calculation of rSO2, the corresponding trace shows periods with no or very low variability. Similarly, both oxyhaemoglobin and deoxyhaemoglobin display distinct differences in time trends, and no NIRS metric is fully coherent with either ABP, ICP or FV, which can be appreciated both when assessing the raw time trends on the left as well as the spectral analyses shown on the right

89 recordings from 35 moderate to severe TBI patients covering 412 h of artefact-free multimodality monitoring data were analysed. The median age was 45 (IQR 35–55), with 86% being male. 53% were admitted with a GCS of 8 or lower, and 20% had suffered an isolated TBI. At 6 months, 17% had died and 51% had reached a favourable outcome (Glasgow Outcome Scale of 1 or 2). An example of the acquired data (both in its raw form on the left and the corresponding spectrum charts on the right) is shown in Fig. 1.

**Fig. 2:**
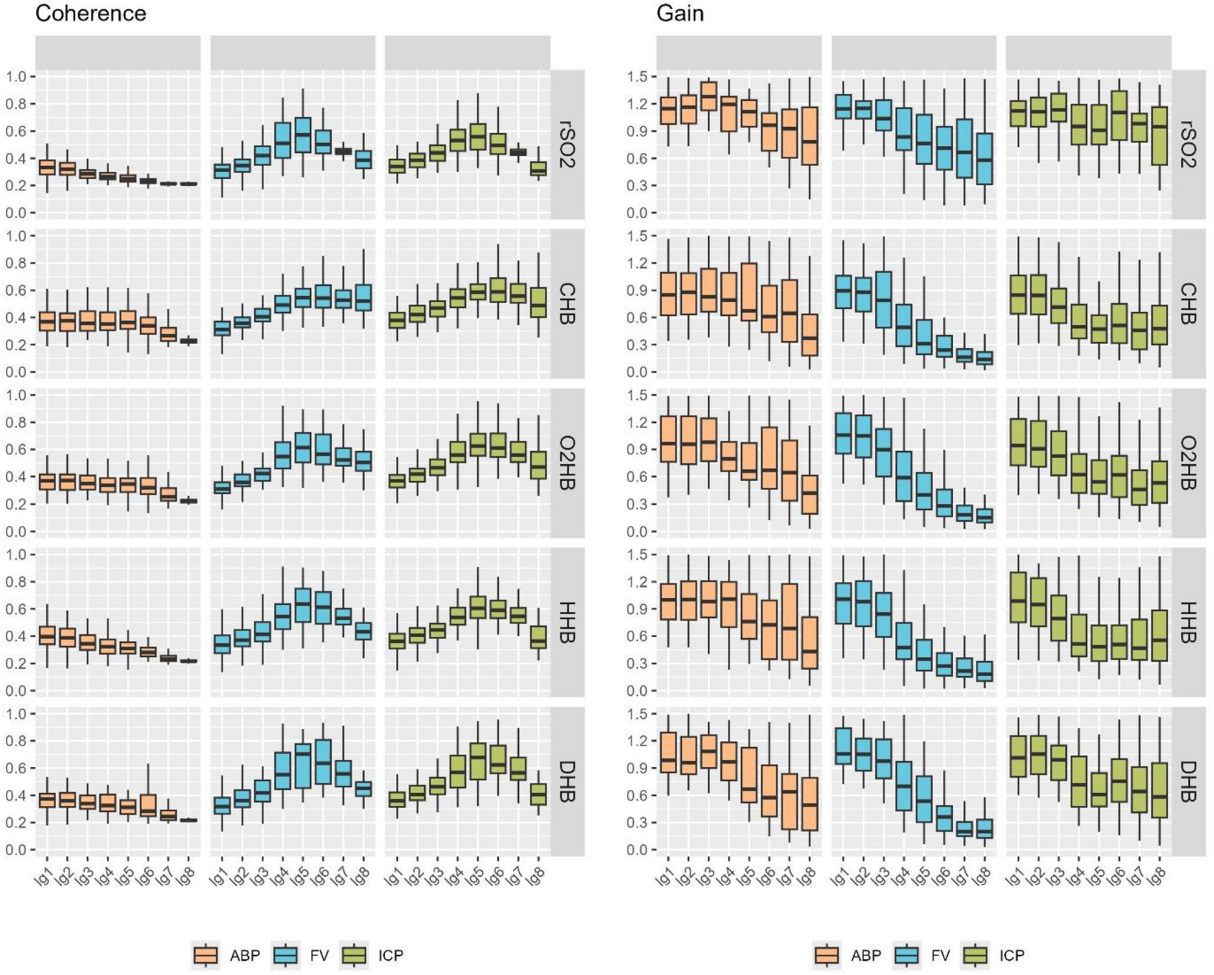
Frequency dependent coherence and gain: logarithmic scale. The levels of coherence (left) and gain (right) dependent on NIRS metric and scale (0.001–0.5 Hz) stratified by the input signal (ABP vs. FV vs. ICP) are shown. Dynamic increases in coherence can be seen peaking around lg4-lg7 for ICP and FV, but not ABP, which displays either stable trends or slight reductions in coherence with higher frequencies. Similarly, gain displays a frequency-specific change with decreasing gain with increasing frequency ranges explored, irrespective of input signal. Overall, these dynamic changes imply a better linear relationship between FV and ICP within the slower frequency ranges, which are affected by cerebrovascular autoregulation.*

**Fig. 3:**
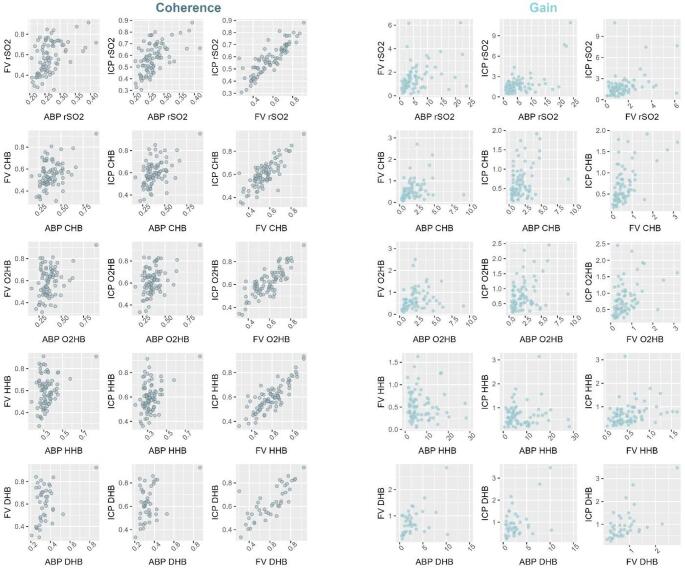
Scatterplots comparing coherence and gain intercorrelations. Within this plot, we were specifically interested in exploring whether there were any clusters of outliers of different coherence and gain combinations, which would imply that there may be specific situations during which these would differ systematically. No such clusters could be identified

The overall patterns of coherence and gain between NIRS metrics and physiological inputs are summarised in Fig. 2, considering the logarithmic scale and Supplement A, considering the linear scale. Coherences to FV and ICP were distinctly frequency dependent and peaked around 0.05 Hz (i.e. logarithmic scale lg5 and linear scale ln1), while coherence to ABP either decreased or remained stable across frequencies, depending on the NIRS metric. In contrast, gain consistently decreased with frequency across all signal combinations. Figure 3 displays the scatter plots comparing coherences and gains between the different inputs (ABP, ICP, FV) and outputs (NIRS metrics) combinations aimed at assessing whether clustered outliers exist. No such distinct clusters could be identified. Additionally, when comparing coherence dependent on the power of slow waves in ABP, significant differences could only be found for the coherence between ABP and all different NIRS derived metrics (*p* < 0.05), while no differences were found for the transmission from FV and ICP (*p* > 0.05).

**Fig. 4:**
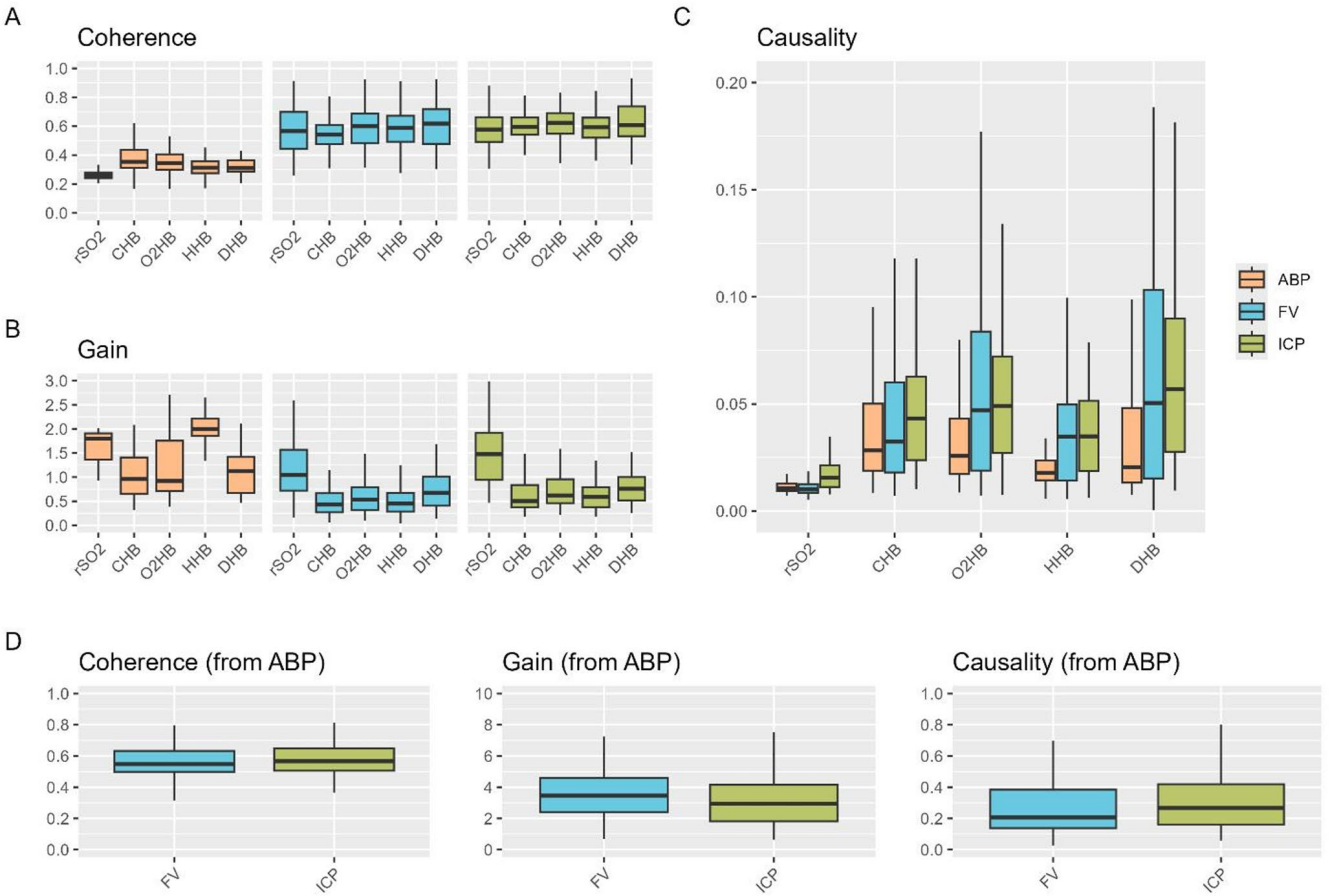
Coherence and gain within the BPL range (0.005 to 0.05 Hz) and Causality. Coherence and gain for the different NIRS derived metrics stratified by input (ABP vs. FV vs. ICP) are shown in panel A and B. Additionally, the level of causality (F statistic) assessed using Granger causality is displayed in panel C. The corresponding statistical analysis is described in Table 2 and Supplement A. Panel D displays the levels of coherence, gain and causality when considering ABP as the input and either FV or ICP as the respective outputs. Overall, the level of coherence was lowest for ABP compared to ICP and FV. The coherence between rSO2 and ABP is particularly low, not reaching the CARNet cutoff of 0.34. The similar levels of coherence between the other NIRS metrics when compared to FV and ICP emphasise that these are affected similarly by the slow waves within the BPL range. Similarly, the gain is similar when considering the haemoglobin measures emphasising that these are affected by incoming slow waves to a similar extent and consequently display the same state of cerebrovascular autoregulation. The levels of causality are distinctly higher for haemoglobin parameters compared to rSO₂ irrespective of the input variable (i.e. ABP, ICP, FV), suggesting that haemoglobin values are more closely associated with preceding variations in these inputs – a necessary condition for accurately assessing cerebrovascular autoregulation

Pairwise comparisons within the BPL range between 0.005 and 0.05 Hz (Fig. 4; Table 2, Supplement B) showed that coherence was significantly higher to FV and ICP than to ABP across all NIRS outputs (*p* < 0.001). Additionally, all haemoglobin metrics displayed stronger coherence to ABP compared to rSO2 (*p* < 0.001, large effect size). Gain was consistent between the haemoglobin parameters when considering FV and ICP as the input, but haemoglobin metrics displayed mostly stronger inhibition of slow wave transmission (i.e. lower gain) compared to rSO2. Granger causality was similar between FV and ICP for the haemoglobin metrics, but only ICP showed consistently higher causality relative to ABP (except for total haemoglobin). Across all analyses, rSO2 consistently showed the lowest coherence and causality with moderate to large effect sizes compared to the different haemoglobin metrics.

**Table 2:** Coherence and gain depending on input stratified by the different NIRS derived measures and Granger causality

		Coherence					
		Input			*p*-value (effect size)		
		ABP	FV	ICP	ABP vs. FV	ABP vs. ICP	FV vs. ICP
**Output**	rSO2	0.26 (0.24, 0.28)	0.57 (0.44, 0.70)	0.58 (0.49, 0.66)	< 0.001 (0.84)	< 0.001 (0.86)	Ns
	CHB	0.35 (0.31, 0.44)	0.54 (0.48, 0.61)	0.60 (0.54, 0.66)	< 0.001 (0.63)	< 0.001 (0.72)	ns
	O2HB	0.34 (0.30, 0.41)	0.60 (0.48, 0.69)	0.62 (0.55, 0.69)	< 0.001 (0.73)	< 0.001 (0.77)	ns
	HHB	0.31 (0.27, 0.36)	0.59 (0.49, 0.67)	0.59 (0.52, 0.66)	< 0.001 (0.79)	< 0.001 (0.82)	ns
	DHB	0.31 (0.29, 0.37)	0.62 (0.47, 0.72)	0.61 (0.53, 0.75)	< 0.001 (0.73)	< 0.001 (0.79)	ns

		Gain					
		Input			*p*-value (effect size)		
		ABP	FV	ICP	ABP vs. FV	ABP vs. ICP	FV vs. ICP
**Output**	rSO2	3.76 (2.17, 6.60)	1.20 (0.78, 1.83)	1.59 (0.99, 2.03)	< 0.001 (0.66)	< 0.001 (0.60)	ns
	CHB	1.43 (0.67, 2.50)	0.44 (0.27, 0.72)	0.50 (0.37, 0.84)	< 0.001 (0.62)	< 0.001 (0.56)	ns
	O2HB	1.86 (0.88, 3.02)	0.54 (0.32, 0.88)	0.64 (0.46, 0.96)	< 0.001 (0.64)	< 0.001 (0.57)	ns
	HHB	5.1 (3.2, 10.8)	0.5 (0.3, 0.7)	0.6 (0.4, 0.8)	< 0.001 (0.85)	< 0.001 (0.85)	ns
	DHB	2.25 (1.10, 3.39)	0.67 (0.39, 1.02)	0.77 (0.52, 1.04)	< 0.001 (0.61)	< 0.001 (0.55)	ns

		Causality					
		Input			*p*-value (effect size)		
		ABP	FV	ICP	ABP vs. FV	ABP vs. ICP	FV vs. ICP
**Output**	rSO2	0.011 (0.009, 0.013)	0.010 (0.008, 0.013)	0.016 (0.011, 0.021)	ns	< 0.001 (0.43)	< 0.001 (0.44)
	CHB	0.03 (0.02, 0.05)	0.03 (0.02, 0.06)	0.04 (0.02, 0.07)	ns	ns	ns
	O2HB	0.03 (0.02, 0.05)	0.05 (0.02, 0.08)	0.05 (0.03, 0.08)	ns	< 0.001 (0.34)	ns
	HHB	0.018 (0.014, 0.024)	0.035 (0.014, 0.050)	0.035 (0.019, 0.052)	0.015 (0.25)	< 0.001 (0.66)	ns
	DHB	0.02 (0.01, 0.06)	0.05 (0.02, 0.10)	0.06 (0.03, 0.11)	ns	< 0.001 (0.38)	ns

The levels of coherence (top) and gain (middle) within the 0.005 to 0.05 Hz frequency band, or causality (bottom) are compared by input and stratified for the different NIRS measures. Wilcoxon rank sum tests were used for comparison with p-values adjusted using the Bonferroni correction. Additionally, the effect size is reported

*Data shown as median (interquartile range); Abbreviations: ABP - arterial blood pressure; CHB - total haemoglobin; DHB - delta haemoglobin; FV - cerebral blood flow velocity; HHB - Deoxyhaemoglobin; ICP - intracranial pressure; ns – not significant; O2HB - Oxyhaemoglobin; rSO2 - regional cerebral oxygen saturation;

The subgroup analyses considering the CARNet ranges are displayed in Supplement C. Overall, the results from the very low frequency range are consistent with the findings considering the BPL range, which it aligns mostly with. Similar trends could be identified for the higher frequency ranges, but coherence dropped distinctly within the high frequency range.

## Discussion

This study explores in detail to what extent the different NIRS metrics capture slow waves found in ABP, FV, or ICP. We specifically focused on the analysis of slow changes to allow the clinician to gauge whether NIRS metrics truly capture changes associated with cerebrovascular autoregulation or reactivity. Our analysis revealed the following:

1. NIRS haemoglobin metrics adequately capture slow waves characteristic of cerebrovascular autoregulation and reactivity.
2. rSO2 demonstrates limited sensitivity for assessing cerebrovascular autoregulation and should, whenever feasible, not be used for evaluating cerebrovascular autoregulation or reactivity.

It is widely accepted that for a monitoring method to be useful, it must accurately measure the process it claims to represent, as even complex measures can be derived from fabricated data without any underlying physiological information [43, 44]. Before using or comparing any correlation-based autoregulation indices derived from NIRS (e.g. cerebral oxygenation index and total haemoglobin index), it is therefore essential to confirm that NIRS reliably captures the slow-wave oscillations associated with cerebrovascular autoregulation. Evaluating this essential aspect was the primary goal of our study. Indeed, a previous study already questioned the agreement between slow waves in ICP, FV, and NIRS metrics and found these agreements to vary dynamically both in time and frequency domain [22]. Both sufficiently high input power [13, 16] (i.e. ABP slow wave power), and sufficient correspondence between the examined signals within the slow frequency range [13] (i.e. coherence, a prerequisite for any cerebrovascular autoregulation estimation), have shown to be imperative for the validity of NIRS derived cerebrovascular autoregulation metrics [32]. We expanded upon these descriptions and provide an in-depth analysis comparing all available NIRS metrics using various different methods to further elucidate these intricate interactions. In our analysis, the coherence threshold suggested by international consensus [32] was only reached by the different haemoglobin metrics, but not rSO2. Our observations align with previous research demonstrating the limited utility of rSO2 for cerebrovascular autoregulation assessment unless large ABP slow waves are induced [16, 45]. Inducing substantial changes in ABP is often impractical if not dangerous in patients requiring continuous cerebrovascular autoregulation monitoring, such as those in intensive care or undergoing anaesthesia during surgical procedures, due to potential adverse effects on other physiological measures. Consequently, continuous approaches must rely on spontaneous ABP fluctuations for estimation [9]. 

The poor synchronisation of rSO2 and ABP within the slow wave range implies it might be affected by other non-linear factors rather than predominantly by cerebrovascular autoregulation-associated changes. This is underscored by both the low coherence as well as low Granger causality identified. These results challenge the assumption that ABP is the primary driver of rSO2 variability, which is a foundational premise for the validity and reliability of autoregulatory indices like the cerebral oxygenation index. Of note, we found sufficiently high coherence between ABP and all the other metrics (FV, ICP, haemoglobin metrics), which confirms that there was overall a sufficient transmission of ABP slow waves. Similarly, previous studies have already identified high coherence between ABP and haemoglobin metrics [46, 47]. The lack of coherence and causality between ABP and rSO2 observed in our study likely resulted from the extensive post-processing applied by the Masimo Root O3 device. This includes low-pass filtering combined with quantisation to 1% resolution, introducing significant non-linear effects. To improve the stability of its time-trend, rSO2 is pre-processed by most of the devices used using different protocols all of which include a level of averaging and low resolution (of maximally 1 Hz) [48, 49] leading to a relevant delay between change in actual saturation and change in rSO2 [50]. In comparison to transcranial Doppler or invasive pressure transducers-based assessments this processing also limits its potential for capturing dynamics as precisely. This is an additional factor that should be taken into account when using COx in research or clinical settings.

Given these findings, an essential question arises regarding whether NIRS should be employed for the assessment of cerebrovascular autoregulation at all. The primary advantage of NIRS, compared to invasive ICP monitoring or transcranial Doppler ultrasound, lies in its ability to monitor a broader patient population, including those who do not meet the criteria for invasive ICP monitoring or who cannot undergo prolonged assessment using transcranial Doppler [27]. It is important to acknowledge that the various parameters commonly used to assess cerebrovascular autoregulation – such as ICP, FV, and NIRS metrics – primarily reflect downstream consequences of the underlying physiological process: changes in arteriolar resistance. This mechanism serves to maintain stable cerebral blood flow, and consequently, cerebral oxygenation. However, a notable delay exists between the initiating event (i.e., a change in blood pressure) and the autoregulatory response [35, 42]. As a result, characteristic slow waves can be observed in these downstream measures, representing dynamic responses to fluctuations in blood pressure [35, 42]. Importantly, each of these metrics can be influenced by additional dynamic factors. For example, ICP is subject to variations in intracranial compliance[51], FV and ICP can be altered by changes in end-tidal CO2 [52, 53], and the various NIRS parameters are influenced by neurovascular coupling and metabolic demand [54, 55]. Consequently, none of these measures perfectly or exclusively quantify the state of cerebrovascular autoregulation or reactivity. However, the high coherence observed between the slow waves in ICP and FV – two widely used and validated proxy measures for cerebrovascular autoregulation and reactivity – and the NIRS haemoglobin metrics supports the notion that these modalities reflect similar underlying changes.

To clarify, our findings do not contradict previously established associations between rSO2, the cerebral oxygenation index, and cerebrovascular autoregulation – particularly those demonstrated under controlled experimental conditions involving substantial induced changes in ABP [16, 45]. Rather, our results emphasise the necessity of considering potential effects introduced by data processing. The use of the rSO2-derived correlation coefficients for monitoring cerebrovascular autoregulation has been previously investigated, primarily through direct comparisons with established indices such as the pressure reactivity [15, 22] or mean flow index [10, 22, 56]. These studies have demonstrated a large variety in intercorrelations; however, most analyses relied on overall average values, with many indices clustering around zero, thereby offering limited insight into the actual state of autoregulation [57, 58]. Additionally, large differences in the time trend of these metrics have been described [7]. 

## Limitations

Despite the large amount of data included, the study was based on a moderate sample size of TBI patients, all recruited from a single centre, with varied types of TBI. This limits generalisability due to potential differences in patient management, disease severity, and TBI subtypes. It is unclear whether these results can be generalised to other diseases. Various confounders may have influenced the findings, including patient-specific haemodynamic signatures, sedation protocols, and medications, as well as disease-specific factors such as the type and extent of intracranial injury. Another limitation of this analysis is its observational design, which restricts findings to associations without allowing for definitive causal inferences. While the interpretations might have been more conclusive if the data had included deliberate stress tests – such as an ABP challenge or ventilator-induced ABP fluctuations [59] – this approach reflects the typical bedside application for surgical or intensive care patients. In these clinical settings, such tests are not routinely feasible; instead, assessments rely on spontaneous fluctuations, as observed in the patient cohort described. Of note, while the methodology of NIRS is relatively similar across different devices, a singular NIRS device with pre-specified (at time unknown and unchangeable) settings and a sampling frequency of 1 Hz was used. Since we were most interested in changes within the slow wave range (i.e. 0.005 and 0.05 Hz), the sampling frequency of 1 Hz (and the corresponding Nyquist limit of 0.5 Hz) does not affect the main conclusions, however, could have affected the estimates within the high frequency ranges (i.e. above 0.2 Hz). Finally, while coherence and causality are great metrics to assess internal consistency and cause and effect respectively their assessment does not in itself indicate the capacity to evaluate cerebral autoregulation. However, a recent study using hemoglobin and rSO_2_-based indices has shown that hemoglobin metrics are better correlated to PRx compared to rSO2-based metric (COx) [24].

### Clinical implications

Our findings suggest that haemoglobin concentration measurements are preferable to rSO2 for assessing cerebrovascular autoregulation and reactivity, as they more accurately capture the characteristic physiological slow changes. In contrast, rSO2 demonstrated overall poor reliability, raising concerns about its validity in evaluating cerebrovascular autoregulation. We did not assess the correlation between NIRS-derived correlation coefficients and well-established measures such as the pressure reactivity index or the mean flow index, as our primary objective was to first determine whether the assumptions necessary for calculating these coefficients were met. However, these correlations could readily be explored, provided that relevant data are collected and processed at the bedside using platforms such as the Moberg CNS Monitor or ICM+. Whenever possible haemoglobin-derived correlation coefficients, such as the total haemoglobin index, should be used for non-invasive NIRS based assessment of cerebrovascular autoregulation or reactivity. Alternatively, to enhance the robustness of cerebrovascular autoregulation assessments derived from rSO2, additional measures such as coherence or power criteria should be incorporated.

## Conclusion

The various NIRS metrics capture slow waves associated with cerebrovascular autoregulation. The changes in haemoglobin concentrations are most suitable for assessing cerebrovascular autoregulation non-invasively. Conversely, rSO2 has a limited value for capturing spontaneously occurring slow waves and consequently cerebrovascular autoregulation monitoring using derived correlation coefficients.

## Supplementary Information

Below is the link to the electronic supplementary material.

Supplementary Material 1

## Data Availability

Data used in this study can be made available upon request.

## Abbreviations

ABP: Arterial blood pressure.
CARNet: Cerebral Autoregulation Research Network
CHB: Total haemoglobin
CPP: Cerebral perfusion pressure
DHB: Delta haemoglobin
FV: Cerebral blood flow velocity
HHB: Deoxyhaemoglobin
ICP: Intracranial pressure
IQR: Interquartile range
NIRS: Near infrared spectroscopy
O2HB: Oxyhaemoglobin
rSO2: Regional cerebral oxygen saturation
TBI: Traumatic brain injury
