# Supplementary material for: On the utility of near-infrared spectroscopy-derived measures for assessing cerebrovascular autoregulation: results from an observational cohort study
Source: J Clin Monit Comput. 2025 Dec 15;40(2):333–44. doi: 10.1007/s10877-025-01399-4 (PMC13053586; doi:10.1007/s10877-025-01399-4)
Supplement: Supplementary file 1 — Supplementary Material 1 [file 10877_2025_1399_MOESM1_ESM.docx]

**Supplement A - Frequency dependent coherence and gain: linear scale*.** The levels of coherence (left) and gain (right) dependent on NIRS metric and scale (0.001 – 0.5 Hz) stratified by the input signal (ABP vs. FV vs. ICP) are shown. In comparison to Figure 2 in the main manuscript, a linear scale is used which allows to focus on the higher frequency bands. The frequency band corresponding to the BPL range lies within ln1 which also displays distinctly higher coherence and gain values in comparison to higher frequency bands.


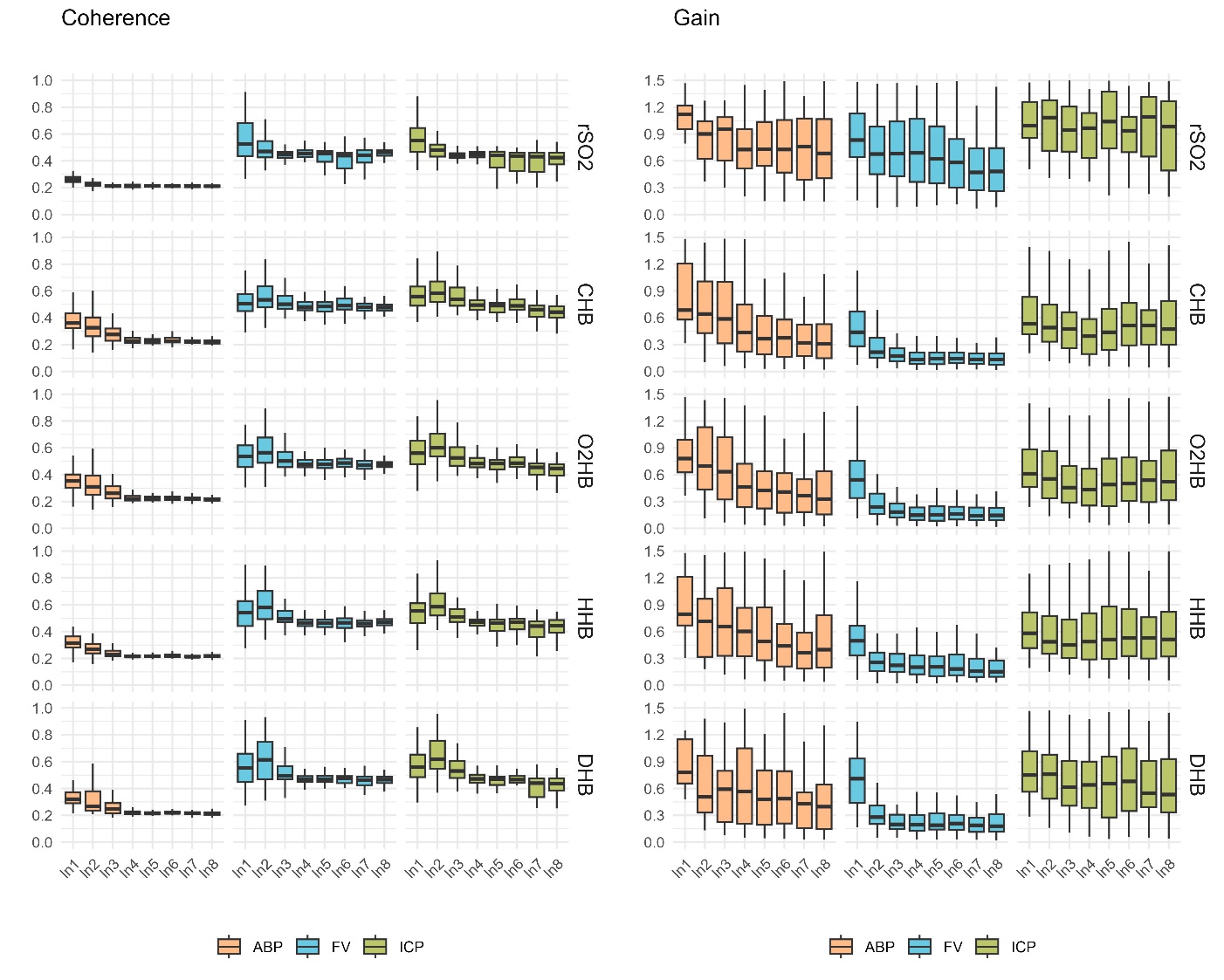
*Abbreviations: ABP: arterial blood pressure; CHB: total haemoglobin; DHB: delta haemoglobin; FV: cerebral blood flow velocity; HHB: deoxyhaemoglobin; ICP: intracranial pressure; ln – linear scale; O2HB: oxyhaemoglobin; rSO2: regional cerebral oxygen saturation;

**Supplement B - Coherence and gain depending on output stratified by the different inputs.** The levels of coherence and gain are compared by output (i.e. NIRS derived metrics) and stratified for the different input measures to assess which NIRS measures has highest coherence or gain associated with either input. Wilcoxon rank sum tests were used for comparison with p-values adjusted using the Bonferroni correction. Additionally, the effect size is reported.*

*Abbreviations: ABP: arterial blood pressure; CHB: total haemoglobin; DHB: delta haemoglobin; FV: cerebral blood flow velocity; HHB: Deoxyhaemoglobin; ICP: intracranial pressure; ns – not significant; O2HB: Oxyhaemoglobin; rSO2: regional cerebral oxygen saturation;

| **Brain Physics Lab Range (0.005 to 0.05 Hz)** | **Coherence: p-values (effect size)** | | | | | | | | | | | |
| --- | --- | --- | --- | --- | --- | --- | --- | --- | --- | --- | --- | --- |
|  |  | rSO2 vs. CHB | rSO2 vs. O2HB | rSO2 vs. HHB | rSO2 vs. DHB | CHB vs. O2HB | CHB vs. HHB | CHB vs. DHB | O2HB vs. HHB | O2HB vs. DHB | HHB vs. DHB |  |
|  | ABP | <0.001 (0.67) | <0.001 (0.63) | <0.001 (0.51) | <0.001 (0.52) | ns | 0.001 (0.31) | ns | ns | ns | ns |  |
|  | FV | ns | ns | ns | ns | ns | ns | ns | ns | ns | ns |  |
|  | ICP | ns | ns | ns | ns | ns | ns | ns | ns | ns | ns |  |
|  |  | | | | | | | | | | | |
|  | **Gain: p-values (effect size)** | | | | | | | | | | | |
|  |  | rSO2 vs. CHB | rSO2 vs. O2HB | rSO2 vs. HHB | rSO2 vs. DHB | CHB vs. O2HB | CHB vs. HHB | CHB vs. DHB | O2HB vs. HHB | O2HB vs. DHB | HHB vs. DHB |  |
|  | ABP | <0.001  (0.56) | <0.001  (0.47) | ns | 0.001  (0.36) | ns | <0.001  (0.67) | ns | <0.001 | ns | <0.001 |  |
|  | FV | <0.001 (0.57) | <0.001 (0.50) | <0.001 (0.61) | <0.001 (0.37) | ns | ns | ns | ns | ns | ns |  |
|  | ICP | <0.001 (0.68) | <0.001 (0.61) | <0.001 (0.69) | <0.001 (0.48) | ns | ns | ns | ns | ns | ns |  |

**Supplement C - Coherence and gain within the CARNet ranges.** Coherence and gain stratified by the CARNet ranges for the different NIRS derived metrics stratified by input (ABP vs. FV vs. ICP) are shown in panel A and B. The corresponding statistical analyses are described in the tables below. The specific frequency ranges are: Very low frequency 0.02 to 0.07 Hz; Low frequency 0.07 to 0.2 Hz; High frequency 0.2 to 0.5 Hz.


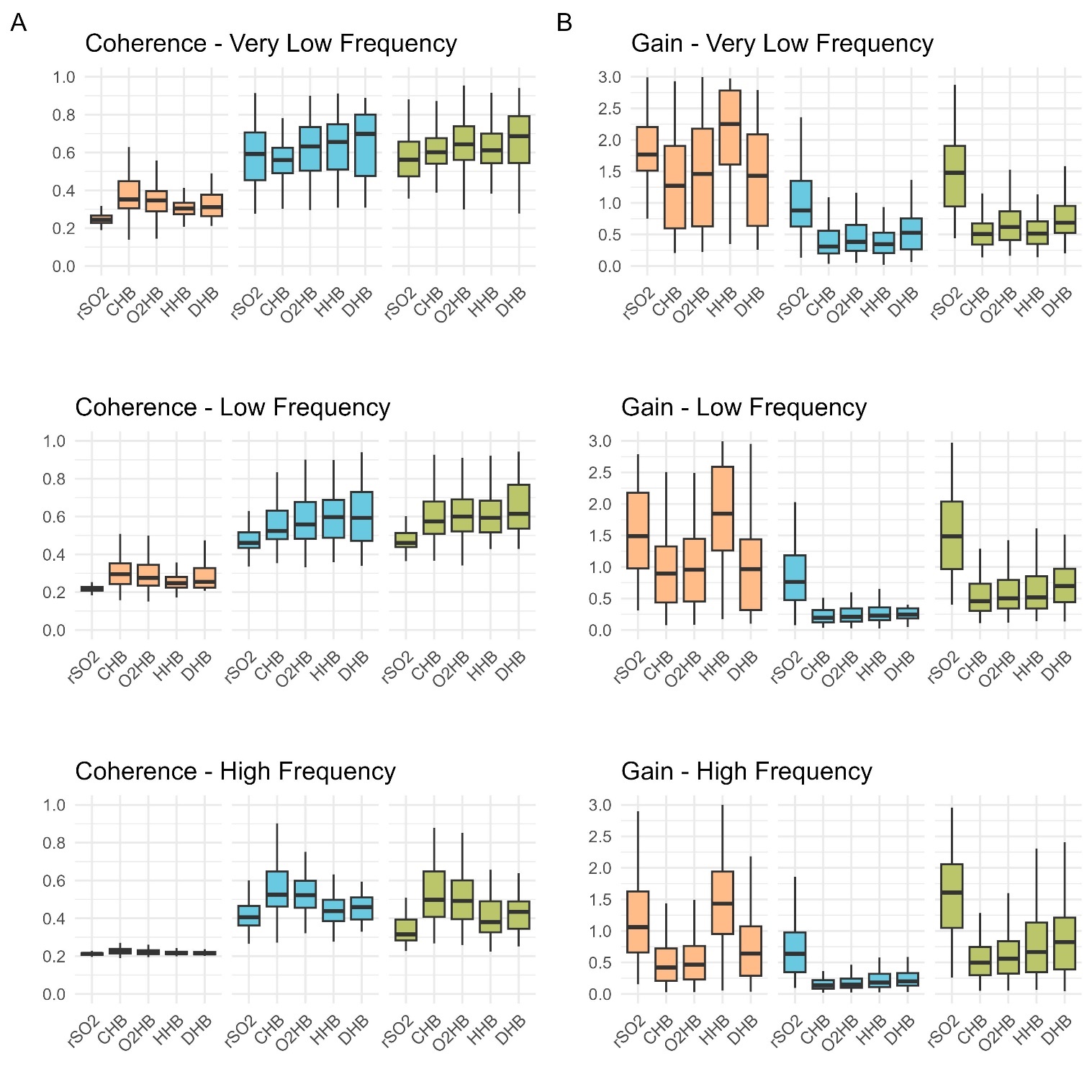
*Abbreviations: ABP: arterial blood pressure; CHB: total haemoglobin; DHB: delta haemoglobin; FV: cerebral blood flow velocity; HHB: Deoxyhaemoglobin; ICP: intracranial pressure; ns – not significant; O2HB: Oxyhaemoglobin; rSO2: regional cerebral oxygen saturation;

**Table 1. Coherence and gain depending on input stratified by the different NIRS derived measures.** The levels of coherence and gain are displayed stratified by the CARNet frequency ranges. Wilcoxon rank sum tests were used for comparison with p-values adjusted using the Bonferroni correction. Additionally, the effect size is reported using superscripts using “^s^”, “^m^”, “^l^”, according to according to Cohen's conventional thresholds (small ≥ 0.1, moderate ≥ 0.3, large ≥ 0.5).*

* Data shown as median (interquartile range); Abbreviations: ABP: arterial blood pressure; CHB: total haemoglobin; DHB: delta haemoglobin; FV: cerebral blood flow velocity; HHB: Deoxyhaemoglobin; ICP: intracranial pressure; ns – not significant; O2HB: Oxyhaemoglobin; rSO2: regional cerebral oxygen saturation;

| **Very Low Frequency Range (0.02 to 0.07 Hz)** | |  | |  | | **Coherence** | | | | | | | | |
| --- | --- | --- | --- | --- | --- | --- | --- | --- | --- | --- | --- | --- | --- | --- |
|  |  |  | |  | | **Input** | | | | | | **p-value (Bonferroni adjusted)** | | |
|  |  |  | |  | | ABP | | FV | | ICP | | ABP vs. FV | ABP vs. ICP | FV vs. ICP |
|  |  | **Output** | | rSO2 | | 0.24 (0.23, 0.27) | | 0.59 (0.45, 0.71) | | 0.56 (0.47, 0.66) | | <0.001 ^l^ | <0.001 ^l^ | ns |
|  |  |  |  | CHB | | 0.35 (0.31, 0.45) | | 0.56 (0.49, 0.63) | | 0.60 (0.54, 0.68) | | <0.001 ^l^ | <0.001 ^l^ | ns |
|  |  |  |  | O2HB | | 0.35 (0.29, 0.40) | | 0.63 (0.50, 0.73) | | 0.64 (0.56, 0.74) | | <0.001 ^l^ | <0.001 ^l^ | ns |
|  |  |  |  | HHB | | 0.30 (0.27, 0.34) | | 0.66 (0.51, 0.75) | | 0.61 (0.54, 0.70) | | <0.001 ^l^ | <0.001 ^l^ | ns |
|  |  |  |  | DHB | | 0.31 (0.26, 0.38) | | 0.70 (0.47, 0.82) | | 0.69 (0.54, 0.80) | | <0.001 ^l^ | <0.001 ^l^ | ns |
|  |  |  | |  | | **Gain** | | | | | | | | |
|  |  |  | |  | | **Input** | | | | | | **p-value (Bonferroni adjusted)** | | |
|  |  |  | |  | | ABP | | FV | | ICP | | ABP vs. FV | ABP vs. ICP | FV vs. ICP |
|  |  | **Output** | | rSO2 | | 4.37 (2.15, 7.69) | | 0.97 (0.64, 1.50) | | 1.54 (1.05, 2.17) | | <0.001 ^l^ | <0.001 ^l^ | 0.002 ^m^ |
|  |  |  |  | CHB | | 1.47 (0.66, 2.51) | | 0.31 (0.20, 0.56) | | 0.51 (0.33, 0.69) | | <0.001 ^l^ | <0.001 ^l^ | 0.006 ^s^ |
|  |  |  |  | O2HB | | 1.91 (0.83, 2.84) | | 0.38 (0.24, 0.66) | | 0.62 (0.41, 0.87) | | <0.001 ^l^ | <0.001 ^l^ | 0.01 ^s^ |
|  |  |  |  | HHB | | 4.9 (3.0, 10.8) | | 0.3 (0.2, 0.5) | | 0.5 (0.3, 0.7) | | <0.001 ^l^ | <0.001 ^l^ | 0.004 ^m^ |
|  |  |  |  | DHB | | 2.08 (1.18, 3.51) | | 0.53 (0.26, 0.76) | | 0.69 (0.52, 0.96) | | <0.001 ^l^ | <0.001 ^l^ | ns |
|  | |  | |  | |  | |  | |  | |  |  |  |
| **Low Frequency Range (0.07 to 0.2 Hz)** |  | |  | | **Coherence** | | | | | | | | | |
|  |  | |  | | **Input** | | | | | | **p-value (Bonferroni adjusted)** | | | |
|  |  | |  | | ABP | | FV | | ICP | | ABP vs. FV | | ABP vs. ICP | FV vs. ICP |
|  | **Output** | | rSO2 | | 0.22 (0.21, 0.23) | | 0.46 (0.43, 0.52) | | 0.46 (0.44, 0.51) | | <0.001 ^l^ | | <0.001 ^l^ | ns |
|  |  |  | CHB | | 0.30 (0.24, 0.35) | | 0.52 (0.48, 0.63) | | 0.57 (0.51, 0.68) | | <0.001 ^l^ | | <0.001 ^l^ | ns |
|  |  |  | O2HB | | 0.28 (0.23, 0.34) | | 0.56 (0.48, 0.68) | | 0.60 (0.52, 0.69) | | <0.001 ^l^ | | <0.001 ^l^ | ns |
|  |  |  | HHB | | 0.25 (0.22, 0.28) | | 0.60 (0.49, 0.69) | | 0.59 (0.52, 0.69) | | <0.001 ^l^ | | <0.001 ^l^ | ns |
|  |  |  | DHB | | 0.25 (0.22, 0.34) | | 0.59 (0.47, 0.74) | | 0.61 (0.53, 0.77) | | <0.001 ^l^ | | <0.001 ^l^ | ns |
|  |  | |  | | **Gain** | | | | | | | | | |
|  |  | |  | | **Input** | | | | | | **p-value (Bonferroni adjusted)** | | | |
|  |  | |  | | ABP | | FV | | ICP | | ABP vs. FV | | ABP vs. ICP | FV vs. ICP |
|  | **Output** | | rSO2 | | 4.16 (1.94, 7.84) | | 0.77 (0.48, 1.23) | | 1.91 (1.13, 3.01) | | <0.001 ^l^ | | <0.001 ^m^ | <0.001 ^l^ |
|  |  |  | CHB | | 1.10 (0.53, 1.78) | | 0.19 (0.12, 0.32) | | 0.46 (0.30, 0.73) | | <0.001 ^l^ | | <0.001 ^m^ | <0.001 ^l^ |
|  |  |  | O2HB | | 1.17 (0.53, 1.97) | | 0.21 (0.13, 0.34) | | 0.50 (0.34, 0.80) | | <0.001 ^l^ | | <0.001 ^m^ | <0.001 ^l^ |
|  |  |  | HHB | | 4.3 (2.2, 10.2) | | 0.2 (0.2, 0.4) | | 0.5 (0.3, 0.9) | | <0.001 ^l^ | | <0.001 ^l^ | 0.02 ^l^ |
|  |  |  | DHB | | 1.20 (0.50, 3.64) | | 0.25 (0.18, 0.35) | | 0.70 (0.44, 0.97) | | <0.001 ^l^ | | 0.009 ^m^ | <0.001 ^l^ |

**Table 1. continued.**

| **High Frequency Range (0.2-0.5 Hz)** |  |  | **Coherence** | | | | | |
| --- | --- | --- | --- | --- | --- | --- | --- | --- |
|  |  |  | **Input** | | | **p-value (Bonferroni adjusted)** | | |
|  |  |  | ABP | FV | ICP | ABP vs. FV | ABP vs. ICP | FV vs. ICP |
|  | **Output** | rSO2 | 0.21 (0.21, 0.22) | 0.41 (0.36, 0.47) | 0.32 (0.28, 0.39) | <0.001 ^l^ | <0.001 ^l^ | <0.001 ^m^ |
|  |  | CHB | 0.22 (0.21, 0.24) | 0.53 (0.46, 0.65) | 0.50 (0.41, 0.65) | <0.001 ^l^ | <0.001 ^l^ | ns |
|  |  | O2HB | 0.22 (0.21, 0.23) | 0.52 (0.46, 0.60) | 0.49 (0.40, 0.60) | <0.001 ^l^ | <0.001 ^l^ | ns |
|  |  | HHB | 0.22 (0.21, 0.22) | 0.44 (0.38, 0.50) | 0.38 (0.33, 0.49) | <0.001 ^l^ | <0.001 ^l^ | ns |
|  |  | DHB | 0.21 (0.21, 0.22) | 0.46 (0.39, 0.51) | 0.43 (0.34, 0.50) | <0.001 ^l^ | <0.001 ^l^ | ns |
|  |  |  | **Gain** | | | | | |
|  |  |  | **Input** | | | **p-value (Bonferroni adjusted)** | | |
|  |  |  | ABP | FV | ICP | ABP vs. FV | ABP vs. ICP | FV vs. ICP |
|  | **Output** | rSO2 | 2.11 (0.93, 3.78) | 0.64 (0.34, 0.98) | 2.08 (1.30, 4.04) | <0.001 ^l^ | ns | <0.001 ^l^ |
|  |  | CHB | 0.42 (0.21, 0.76) | 0.14 (0.08, 0.22) | 0.50 (0.30, 0.75) | <0.001 ^l^ | ns | <0.001 ^l^ |
|  |  | O2HB | 0.48 (0.25, 0.83) | 0.15 (0.10, 0.25) | 0.57 (0.32, 0.87) | <0.001 ^l^ | ns | <0.001 ^l^ |
|  |  | HHB | 1.98 (1.18, 4.07) | 0.18 (0.11, 0.32) | 0.67 (0.34, 1.15) | <0.001 ^l^ | <0.001 ^l^ | <0.001 ^l^ |
|  |  | DHB | 0.67 (0.29, 1.11) | 0.20 (0.13, 0.33) | 0.82 (0.38, 1.25) | <0.001 ^m^ | ns | <0.001 ^l^ |

| **Very Low Frequency Range** | **Coherence: p-values (Bonferroni adjusted)** | | | | | | | | | | |
| --- | --- | --- | --- | --- | --- | --- | --- | --- | --- | --- | --- |
|  |  | rSO2 vs. CHB | rSO2 vs. O2HB | rSO2 vs. HHB | rSO2 vs. DHB | CHB vs. O2HB | CHB vs. HHB | CHB vs. DHB | O2HB vs. HHB | O2HB vs. DHB | HHB vs. DHB |
|  | ABP | <0.001 ^l^ | <0.001 ^l^ | <0.001 ^l^ | <0.001 ^l^ | ns | <0.001 ^m^ | ns | 0.01 ^s^ | ns | ns |
|  | FV | ns | ns | ns | ns | ns | ns | ns | ns | ns | ns |
|  | ICP | ns | ns | ns | ns | ns | ns | ns | ns | ns | ns |
|  |  | | | | | | | | | | |
|  | **Gain: p-values (Bonferroni adjusted)** | | | | | | | | | | |
|  |  | rSO2 vs. CHB | rSO2 vs. O2HB | rSO2 vs. HHB | rSO2 vs. DHB | CHB vs. O2HB | CHB vs. HHB | CHB vs. DHB | O2HB vs. HHB | O2HB vs. DHB | HHB vs. DHB |
|  | ABP | <0.001 ^l^ | <0.001 ^m^ | ns | 0.004 ^m^ | ns | <0.001 ^l^ | ns | <0.001 ^l^ | ns | <0.001 ^m^ |
|  | FV | <0.001 ^l^ | <0.001 ^l^ | <0.001 ^l^ | <0.001 ^m^ | ns | ns | ns | ns | ns | ns |
|  | ICP | <0.001 ^l^ | <0.001 ^l^ | <0.001 ^l^ | <0.001 ^l^ | ns | ns | ns | ns | ns | Ns |
|  | | | | | | | | | | | |
| **Low Frequency Range** | **Coherence: p-values (Bonferroni adjusted)** | | | | | | | | | | |
|  |  | rSO2 vs. CHB | rSO2 vs. O2HB | rSO2 vs. HHB | rSO2 vs. DHB | CHB vs. O2HB | CHB vs. HHB | CHB vs. DHB | O2HB vs. HHB | O2HB vs. DHB | HHB vs. DHB |
|  | ABP | <0.001 ^l^ | <0.001 ^l^ | <0.001 ^l^ | <0.001 ^l^ | ns | 0.001 ^m^ | ns | ns | ns | ns |
|  | FV | <0.001 ^m^ | <0.001 ^m^ | <0.001 ^m^ | <0.001 ^m^ | ns | ns | ns | ns | ns | ns |
|  | ICP | <0.001 ^l^ | <0.001 ^l^ | <0.001 ^l^ | <0.001 ^l^ | ns | ns | ns | ns | ns | ns |
|  |  | | | | | | | | | | |
|  | **Gain: p-values (Bonferroni adjusted)** | | | | | | | | | | |
|  |  | rSO2 vs. CHB | rSO2 vs. O2HB | rSO2 vs. HHB | rSO2 vs. DHB | CHB vs. O2HB | CHB vs. HHB | CHB vs. DHB | O2HB vs. HHB | O2HB vs. DHB | HHB vs. DHB |
|  | ABP | <0.001 ^l^ | <0.001 ^l^ | ns | <0.001 ^m^ | ns | <0.001 ^l^ | ns | <0.001 ^l^ | ns | <0.001 ^m^ |
|  | FV | <0.001 ^l^ | <0.001 ^l^ | <0.001 ^l^ | <0.001 ^l^ | ns | ns | ns | ns | ns | ns |
|  | ICP | <0.001 ^l^ | <0.001 ^l^ | <0.001 ^l^ | <0.001 ^l^ | ns | ns | ns | ns | ns | ns |
|  | | | | | | | | | | | |
| **High Frequency Range** | **Coherence: p-values (Bonferroni adjusted)** | | | | | | | | | | |
|  |  | rSO2 vs. CHB | rSO2 vs. O2HB | rSO2 vs. HHB | rSO2 vs. DHB | CHB vs. O2HB | CHB vs. HHB | CHB vs. DHB | O2HB vs. HHB | O2HB vs. DHB | HHB vs. DHB |
|  | ABP | <0.001 ^m^ | 0.001 ^m^ | ns | ns | ns | 0.006 ^m^ | 0.03 ^m^ | ns | ns | ns |
|  | FV | ns | ns | ns | ns | ns | ns | ns | ns | ns | ns |
|  | ICP | ns | ns | ns | ns | ns | ns | ns | ns | ns | ns |
|  |  | | | | | | | | | | |
|  | **Gain: p-values (Bonferroni adjusted)** | | | | | | | | | | |
|  |  | rSO2 vs. CHB | rSO2 vs. O2HB | rSO2 vs. HHB | rSO2 vs. DHB | CHB vs. O2HB | CHB vs. HHB | CHB vs. DHB | O2HB vs. HHB | O2HB vs. DHB | HHB vs. DHB |
|  | ABP | <0.001 ^l^ | <0.001 ^l^ | ns | <0.001 ^m^ | ns | <0.001 ^l^ | ns | <0.001 ^l^ | ns | <0.001 ^l^ |
|  | FV | <0.001 ^l^ | <0.001 ^l^ | <0.001 ^l^ | <0.001 ^l^ | ns | ns | ns | ns | ns | ns |
|  | ICP | <0.001 ^l^ | <0.001 ^l^ | <0.001 ^l^ | <0.001 ^l^ | ns | ns | ns | ns | ns | ns |

**Table 2. Coherence and gain depending on output stratified by the different inputs.** The statistical analysis stratified by the CARNet frequency ranges is shown. Wilcoxon rank sum tests were used for comparison with p-values adjusted using the Bonferroni correction. Effect size is reported using superscripts using “^s^”, “^m^”, “^l^”, according to according to Cohen's conventional thresholds (small ≥ 0.1, moderate ≥ 0.3, large ≥ 0.5).*

* Data shown as median (interquartile range); Abbreviations: ABP: arterial blood pressure; CHB: total haemoglobin; DHB: delta haemoglobin; FV: cerebral blood flow velocity; HHB: Deoxyhaemoglobin; ICP: intracranial pressure; ns – not significant; O2HB: Oxyhaemoglobin; rSO2: regional cerebral oxygen saturation;
